# Chemopreventive Effects of Bioactive Peptides Derived from Black Soldier Fly Larvae Protein Hydrolysates in a Rat Model of Early-Stage Colorectal Carcinogenesis

**DOI:** 10.3390/ijms26135955

**Published:** 2025-06-20

**Authors:** Kwanchanok Praseatsook, Arpamas Vachiraarunwong, Kenji Sato, Sivamoke Dissook, Hideki Wanibuchi, Sirinya Taya, Rawiwan Wongpoomchai, Pornngarm Dejkriengkraikul, Min Gi, Supachai Yodkeeree

**Affiliations:** 1 Department of Biochemistry, Faculty of Medicine, Chiang Mai University, Chiang Mai 50200, Thailand; kwanchanok_pa@cmu.ac.th (K.P.); sivamoke.dis@cmu.ac.th (S.D.); rawiwan.wong@cmu.ac.th (R.W.); pornngarm.d@cmu.ac.th (P.D.); 2 Department of Environmental Risk Assessment, Osaka Metropolitan University Graduate School of Medicine, Osaka 545-8585, Japan; arpamas.vachi@omu.ac.jp (A.V.); wani@omu.ac.jp (H.W.); 3 Division of Applied Biosciences, Graduate School of Agriculture, Kyoto University, Kyoto 606-8502, Japan; sato.kenji.7x@kyoto-u.ac.jp; 4 Functional Food Research Unit, Multidisciplinary Research Institute, Chiang Mai University, Chiang Mai 50200, Thailand; sirinya.t@cmu.ac.th; 5 Anticarcinogenesis and Apoptosis Research Cluster, Faculty of Medicine, Chiang Mai University, Chiang Mai 50200, Thailand

**Keywords:** black soldier fly larvae, insect protein hydrolysates, spray drying, freeze drying, colon cancer, gut microbiota, short-chain fatty acids

## Abstract

Bioactive peptides from black soldier fly larvae (BSFL) protein hydrolysates have gained attention for their health-promoting properties. Our previous study demonstrated the chemopreventive potential of BSFL hydrolysates prepared with Alcalase (ASBP-AH) in colon cancer cells; their in vivo efficacy has not been fully elucidated. This study evaluated the chemopreventive effects of ASBP-AH, processed by spray-drying (ASBP-AHS) or freeze-drying (ASBP-AHF), in a diethylnitrosamine (DEN) and 1,2-dimethylhydrazine (DMH)-induced rat model of early-stage colorectal carcinogenesis. Oral administration of ASBP-AHS or ASBP-AHF significantly reduced aberrant crypt foci (ACF) and downregulated PCNA, COX-2, and NF-κB expression, without affecting apoptosis. Furthermore, both treatments restored microbial species richness and shifted gut microbial diversity disrupted by carcinogen exposure. ASBP-AHS specifically enriched short-chain fatty acid (SCFA)-producing bacteria, while ASBP-AHF favored anti-inflammatory microbial signatures. Likewise, correlation analysis revealed positive associations between microbial changes and SCFA levels, particularly with ASBP-AHS. Peptidomic profiling identified identical peptides in both hydrolysates, including stable pyroglutamyl-containing sequences with potential anti-inflammatory and microbiota-modulating effects. These findings support the in vivo chemopreventive potential of ASBP-AH and its promise as a functional food ingredient for promoting gut health and reducing colorectal cancer risk.

## 1. Introduction

Colorectal cancer (CRC) is a major cause of cancer-related mortality worldwide and is driven by multiple factors, including diet, genetic mutations, inflammation, and gut microbiota imbalance. Risk factors such as high intake of red or processed meats, low fiber consumption, alcohol use, and exposure to carcinogens contribute to CRC progression. [1,2]. Current CRC treatments include surgery, chemotherapy, and targeted therapies, selected based on tumor stage and molecular features. However, chemotherapeutic agents often cause severe toxicity, adverse effects, and drug resistance due to their impact on various normal organ systems, not just cancerous tissues [3,4]. Therefore, there is an urgent need to develop alternative therapeutic approaches or novel anti-colorectal cancer and chemo-preventive agents with high efficacy and low toxicity.

Food-derived bioactive peptides have gained attention for their diverse biological activities, including antioxidant, anticancer, anti-inflammatory, and immunomodulatory effects. Their functional properties are influenced by molecular size, amino acid composition, and sequence [5]. Recent research has increasingly highlighted the therapeutic and preventive potential of protein-derived peptides in colon cancer, with numerous in vitro and in vivo studies demonstrating their efficacy in suppressing tumor growth, inducing apoptosis, and modulating cancer-related pathways. For example, peptides from *Gloriosa superba* and lunasin from soybean promote apoptosis via p53 activation and mitochondrial pathways in colon cancer cells [6,7], while uroguanylin inhibits polyp formation in CRC mouse models [8]. These peptides exhibit high selectivity to cancer cells while showing minimal toxicity to normal cells. Their low molecular weight enhances intracellular transport and membrane interactions, leading to cancer cell death [9].

Increasing evidence also implicates gut microbiota dysbiosis in the pathogenesis and progression of CRC. The microbial composition differs significantly between healthy individuals and CRC patients, with dysbiosis contributing to chronic inflammation, immune dysregulation, and production of toxic metabolites [10]. Some food-derived peptides have demonstrated gut-modulating properties. Notably, pyroglutamyl (pGlu)-Leu, a dipeptide derived from wheat gluten hydrolysate, restored the Bacteroidetes-to-Firmicutes balance in a dextran sulfate sodium (DSS)-induced colitis mouse model [11]. Similarly, quinoa protein hydrolysates attenuated azoxymethane (AOM)/DSS-induced colorectal carcinogenesis in mice by modulating gut microbiota and promoting the production of beneficial short-chain fatty acids (SCFAs) [12].

Black soldier fly larvae (*Hermetia illucens*, BSFL) protein has gained increasing attention as a sustainable source of nutrition and functional peptides. Notably, enzymatic hydrolysis using Alcalase has been reported to reduce allergenic proteins in BSFL, supporting its potential safety for use in food applications [13]. Our previous study demonstrated that the alkali-soluble protein extracted from BSFL, when hydrolyzed with Alcalase (ASBP-AH), exhibited significant antioxidant, anti-mutagenic, anti-inflammatory, and anticancer activities in colon cancer cell models under in vitro conditions [14]. However, its chemopreventive potential in vivo remains to be elucidated. In this study, a dual-organ carcinogenicity model using diethylnitrosamine (DEN) and 1,2-dimethylhydrazine (DMH) was employed to assess the effects of ASBP-AH on liver and colon tumor development. This model enables evaluation of test compounds in multiple organs within a single animal over a short period [15]. However, the current study specifically focused on the chemopreventive effect of ASBP-AH on DMH-induced aberrant crypt foci (ACF) in the colon. DMH is a potent carcinogen widely used to induce colorectal tumors in rodent models due to its close resemblance to the pathophysiology of human CRC [16]. Carcinogenesis typically begins at the colonic crypts, with ACF forming as early preneoplastic lesions that can progress to microadenomas, adenomas, and ultimately adenocarcinomas [17]. Additionally, DMH disrupts gut microbial balance, contributing to CRC progression through dysbiosis [18]. Therefore, the present study aims to evaluate the in vivo chemopreventive potential of ASBP-AH in early-stage DMH-induced colorectal carcinogenesis in rats. We investigated its effects on ACF formation, inflammation, cell proliferation, and apoptosis, along with its influence on gut microbiota balance.

## 2. Results

### 2.1. Effect of ASBP, ASBP-AHS, and ASBP-AHF on Physiological Parameters and Liver Function Enzymes

The administration of alkali-soluble Black Soldier Fly larvae protein (ASBP), ASBP-AH prepared by spray-drying (ASBP-AHS), and ASBP-AH prepared by freeze-drying (ASBP-AHF) did not result in significant alterations in body weight, food intake, or water consumption across all treatment groups compared to the DEN + DMH group (Table 1). These findings suggest that the tested samples did not adversely affect general metabolic status or feeding behavior. Additionally, no significant differences were observed in the absolute or relative weights of the liver, kidneys, and spleen among the experimental groups (Table 2), indicating that the treatments did not induce organ hypertrophy or atrophy.

Serum aspartate aminotransferase (AST) and alanine aminotransferase (ALT) levels, key indicators of hepatic injury, remained unchanged following administration of ASBP, ASBP-AHS, or ASBP-AHF alone when compared to the normal saline solution (NSS) group (Table 3), indicating no hepatotoxicity. In contrast, all DEN + DMH-treated groups showed significantly elevated AST and/or ALT levels compared to the NSS group. However, none of the combination treatments—(DEN + DMH) + ASBP, (DEN + DMH) + ASBP-AHS, or (DEN + DMH) + ASBP-AHF—significantly reduced AST or ALT levels relative to the DEN + DMH group. These results indicate that while ASBP and its hydrolysates are not hepatotoxic, they do not exhibit protective effects against DEN + DMH-induced hepatic injury.

### 2.2. Effects of ASBP, ASBP-AHS, and ASBP-AHF on Preneoplastic Lesions in the Colon Tissues of DEN and DMH-Treated Rats

**Figure 1:**
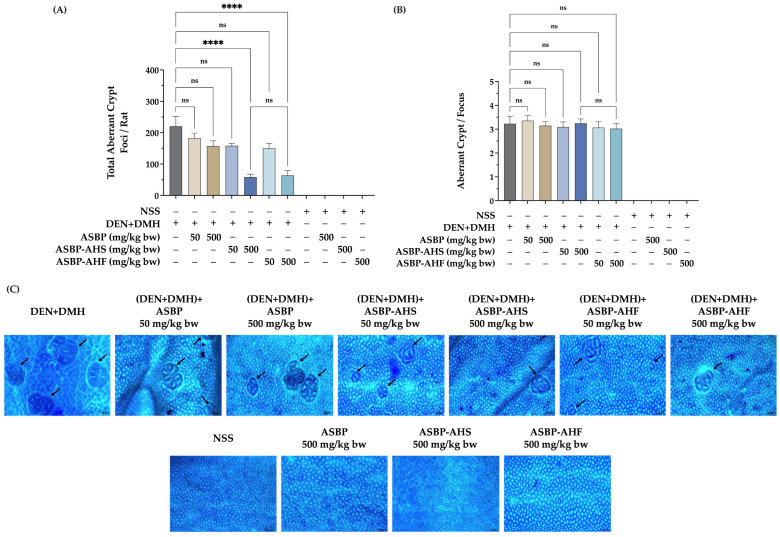
Effects of ASBP, ASBP-AHS, and ASBP-AHF on preneoplastic lesions in DEN- and DMH-induced early-stage colon carcinogenesis in rats. (**A**) Total number of aberrant crypt foci (ACF) per rat. (**B**) Number of aberrant crypts per focus (AC/F) in colon tissues. (**C**) Representative image of ACF (black arrows) in colon tissue at 50× magnification. The statistical analyses were performed using one-way analysis of variance (ANOVA), followed by Tukey’s post hoc test. Significant differences between groups are indicated as **** *p* < 0.0001; ns = not significant. Abbreviations: normal saline solution (NSS); Diethylnitrosamine (DEN); 1,2-dimethylhydrazine (DMH); alkali-soluble BSFL protein (ASBP); alkali-soluble BSFL protein-Alcalase hydrolysate (spray-dried, ASBP-AHS; freeze-dried, ASBP-AHF); aberrant crypt foci (ACF).

In the DMH-induced rat model, ACF are recognized as preneoplastic lesions indicative of the early stages of colorectal carcinogenesis. To evaluate the chemopreventive potential of ASBP, ASBP-AHS, and ASBP-AHF, ACF formation was assessed in DEN + DMH-treated rats. As expected, the DEN + DMH group exhibited a significantly higher number of ACF compared to the NSS control group. Although ASBP at 500 mg/kg body weight reduced ACF formation relative to the DEN + DMH group, the reduction was not statistically significant. In contrast, high-dose administration of ASBP-AHS and ASBP-AHF significantly decreased ACF numbers, indicating a potential protective effect (Figure 1A,C). However, ACF size remained unchanged, with large foci observed across all treatment groups (Figure 1B,C). These findings suggest that high concentrations of ASBP-AHS and ASBP-AHF may suppress early-stage colonic lesions in DEN + DMH-induced carcinogenesis.

### 2.3. Reduction in Proliferating Cell Nuclear Antigen-Positive Cells by ASBP-AHS and ASBP-AHF in Early DEN and DMH-Induced Colon Carcinogenesis

**Figure 2:**
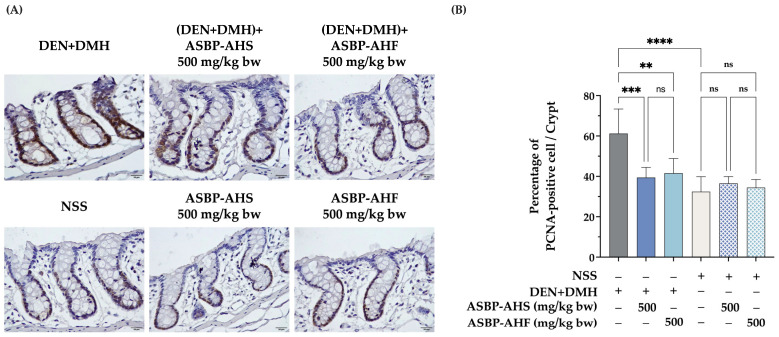
Effects of ASBP-AHS and ASBP-AHF on PCNA expression in colon tissues during DEN + DMH-induced early-stage colon carcinogenesis. (**A**) Representative immunohistochemical images of PCNA-positive cells in colon tissues (500× magnification), with brown staining indicating PCNA expression. (**B**) Quantification of PCNA-positive cells per crypt. The data are presented as mean ± SD. The statistical analyses were performed using one-way analysis of variance (ANOVA), followed by Tukey’s post hoc test. Significant differences between groups are indicated as ** *p* < 0.01, *** *p* < 0.001, **** *p* < 0.0001; ns = not significant. Abbreviations: normal saline solution (NSS); Diethylnitrosamine (DEN); 1,2-dimethylhydrazine (DMH); alkali-soluble BSFL protein (ASBP); alkali-soluble BSFL protein-Alcalase hydrolysate (spray-dried, ASBP-AHS; freeze-dried, ASBP-AHF); proliferating cell nuclear antigen (PCNA).

To further elucidate the mechanisms underlying the protective effects of ASBP-AHS and ASBP-AHF, cell proliferation and apoptosis in colonic tissues were evaluated using proliferating cell nuclear antigen (PCNA) and cleaved caspase-3 immunostaining, respectively. As shown in Figure 2A,B, the DEN + DMH-treated group exhibited a significantly higher percentage of PCNA-positive cells per crypt compared to the NSS control, indicating elevated proliferative activity associated with early carcinogenic processes. Administration of ASBP-AHS or ASBP-AHF at 500 mg/kg body weight significantly reduced the number of PCNA-positive cells induced by DEN + DMH. Notably, no significant difference was observed between ASBP-AHS and ASBP-AHF, suggesting that the drying method did not influence their anti-proliferative properties. In contrast, cleaved caspase-3 staining showed no detectable expression in any group (Figure S1), indicating that the observed suppression of proliferation by ASBP-AHS and ASBP-AHF was not associated with caspase-3-mediated apoptotic cell death.

### 2.4. Suppression of NF-κB and COX-2 Expression by ASBP-AHS and ASBP-AHF in Early DEN and DMH-Induced Colon Carcinogenesis

**Figure 3:**
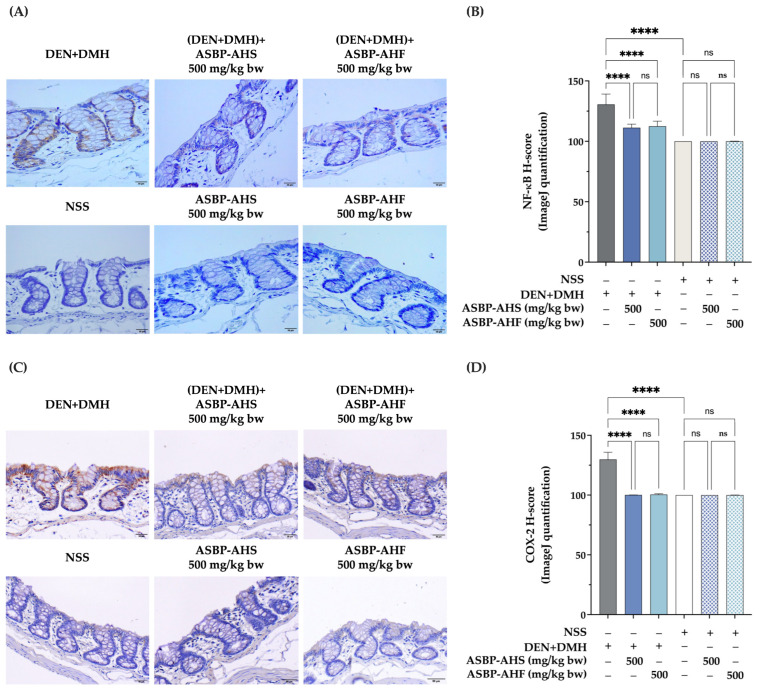
Effects of ASBP-AHS and ASBP-AHF on NF-κB and COX-2 expression in colon tissues during DEN + DMH-induced early-stage colon carcinogenesis. (**A**) Representative immunohistochemical staining of NF-κB in colon tissues at 500× magnification, with brown coloration indicating protein expression. (**B**) H-score quantification of NF-κB expression. (**C**) Representative immunohistochemical staining of COX-2 in colon tissues at 500× magnification, with brown coloration indicating protein expression. (**D**) H-score quantification of COX-2 expression. The data are presented as mean ± SD. The statistical analyses were performed using one-way analysis of variance (ANOVA), followed by Tukey’s post hoc test. Significant differences between groups are indicated as **** *p* < 0.0001; ns = not significant. Abbreviations: normal saline solution (NSS); Diethylnitrosamine (DEN); 1,2-dimethylhydrazine (DMH); alkali-soluble BSFL protein (ASBP); alkali-soluble BSFL protein-Alcalase hydrolysate (spray-dried, ASBP-AHS; freeze-dried, ASBP-AHF).

The effects of ASBP-AHS and ASBP-AHF on inflammation-associated markers were evaluated by assessing nuclear factor kappa B (NF-κB) and cyclooxygenase-2 (COX-2) expression via immunohistochemical staining and histological score (H-score) analysis. As shown in Figure 3A,B, colonic tissues from the DEN + DMH group exhibited increased NF-κB staining intensity and H-scores compared to the NSS control. In contrast, treatment with ASBP-AHS or ASBP-AHF significantly attenuated DEN + DMH-induced NF-κB expression, resulting in intensity levels comparable to those observed in the NSS group. Similarly, both treatments markedly reduced COX-2 expression induced by DEN + DMH, as evidenced by decreased staining intensity and lower H-scores (Figure 3C,D). No significant differences in NF-κB or COX-2 expression were observed between the ASBP-AHS- and ASBP-AHF-treated groups and the NSS group. Collectively, these findings suggest that ASBP-AHS and ASBP-AHF exert anti-inflammatory effects in DEN + DMH-induced early-stage colon carcinogenesis, likely through the suppression of NF-κB and COX-2 signaling pathways.

### 2.5. Modulation of Gut Microbiota by ASBP-AHS and ASBP-AHF in DEN and DMH-Treated Rats

**Figure 4:**
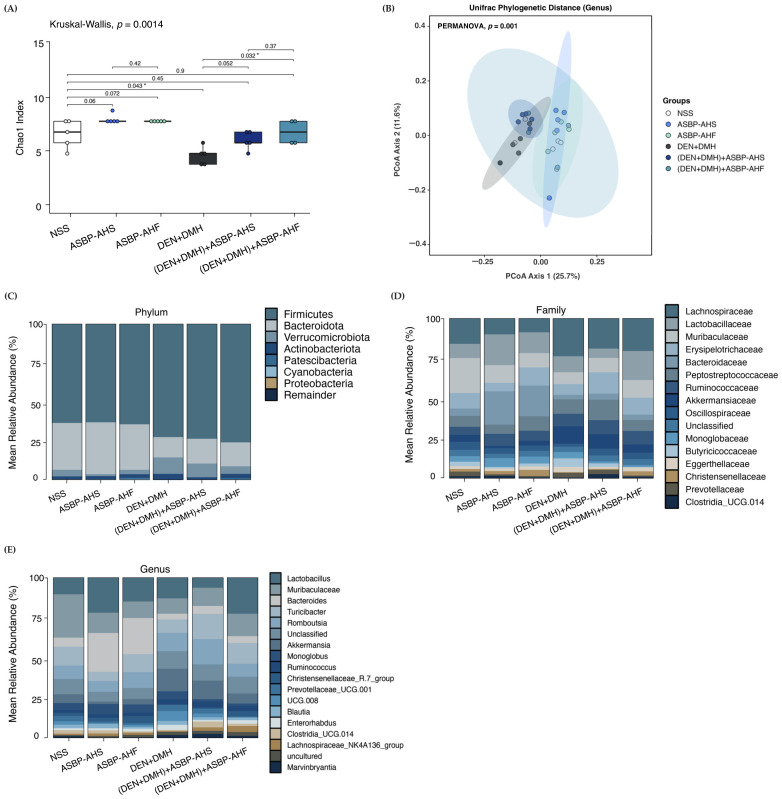
Restorative modulation of gut microbiota by ASBP-AHS and ASBP-AHF following DEN and DMH exposure. (**A**) Alpha diversity assessed using the Chao1 index. (**B**) Beta diversity analysis based on Principal Coordinates Analysis (PCoA) of UniFrac phylogenetic distances at the genus level. (**C**–**E**) Mean relative abundances of gut microbiota at the phylum (**C**), family (**D**), and genus (**E**) levels. Each group included *n* = 5 rats, except the (DEN + DMH) + ASBP-AHF group, which included *n* = 4. The statistical differences among groups were evaluated using the Kruskal–Wallis test, followed by Wilcoxon rank-sum tests for pairwise comparisons. Significant differences between groups are indicated as * *p* < 0.05. Abbreviations: normal saline solution (NSS); Diethylnitrosamine (DEN); 1,2-dimethylhydrazine (DMH); alkali-soluble BSFL protein (ASBP); alkali-soluble BSFL protein-Alcalase hydrolysate (spray-dried, ASBP-AHS; freeze-dried, ASBP-AHF).

The modulatory effects of ASBP-AHS and ASBP-AHF on gut microbiota composition were examined in a DEN- and DMH-induced colorectal carcinogenesis model. Alpha diversity analysis using the Chao1 index revealed a significant reduction in species richness in the DEN + DMH group compared to the NSS group. Post-treatment with ASBP-AHS or ASBP-AHF increased species richness, with ASBP-AHF showing a statistically significant improvement, suggesting a greater potential to restore microbial diversity (Figure 4A). Beta diversity analysis using UniFrac distances demonstrated distinct clustering among groups (PERMANOVA, *p* = 0.001), indicating significant shifts in microbial community structure (Figure 4B). The DEN + DMH group clustered separately from the NSS group, reflecting carcinogen-induced dysbiosis. Notably, the (DEN + DMH) + ASBP-AHS and (DEN + DMH) + ASBP-AHF groups shifted closer to the NSS cluster, with ASBP-AHF showing the greatest resemblance, suggesting a stronger restorative effect. In contrast, ASBP-AHS and ASBP-AHF treatments alone did not significantly alter microbial structure under non-carcinogenic conditions.

The mean relative abundance of gut microbiota at the phylum, family, and genus levels was assessed to characterize microbial alterations associated with early-stage colorectal carcinogenesis and to evaluate the effects of ASBP-AHS and ASBP-AHF interventions. At the phylum level, the NSS group exhibited a balanced microbial profile dominated by *Firmicutes* (63.35%) and *Bacteroidota* (30.09%). Similar distributions were observed in the ASBP-AHS and ASBP-AHF groups, indicating no major alterations under non-carcinogenic conditions. In contrast, the DEN + DMH group displayed increased *Firmicutes* (72.45%), reduced *Bacteroidota* (13.06%), and elevated *Verrucomicrobiota*, reflecting carcinogen-induced dysbiosis. Post-treatment with ASBP-AHS and ASBP-AHF moderately increased *Bacteroidota* (15.95% and 15.35%, respectively) and reduced *Verrucomicrobiota* levels, suggesting partial restoration of microbial balance, although the composition was not fully restored to NSS levels (Figure 4C).

At the family level (Figure 4D), DEN + DMH exposure increased the relative abundance of *Lachnospiraceae* and *Akkermansiaceae*, both associated with impaired gut barrier function and inflammation. In contrast, ASBP-AHS and ASBP-AHF treatments alone increased *Lactobacillaceae* levels to 18.71% and 12.60%, respectively, compared to 8.40% in the NSS group. Notably, ASBP-AHF sustained *Lactobacillaceae* abundance (17.23%) even after carcinogen exposure, compared to the DEN + DMH group (9.66%). *Christensenellaceae,* a family linked to anti-inflammatory and metabolic benefits, was consistently enriched in the ASBP-AHF group under both conditions. Additionally, ASBP-AHS treatment under carcinogenic conditions increased the abundance of *Clostridia_UCG.014*, *Erysipelotrichaceae*, and *Peptostreptococcaceae* compared to the DEN + DMH group, suggesting enhanced microbial restoration and potential immunoregulatory effects.

At the genus level (Figure 4E), ASBP-AHS treatment under non-carcinogenic conditions resulted in the highest relative abundance of *Lactobacillus* (18.71%) and *Bacteroides* (20.32%), followed by the ASBP-AHF and NSS groups. Both genera are key SCFA producers and indicators of gut health. *Muribaculaceae* was reduced in the ASBP-AHS (10.72%) and ASBP-AHF (8.52%) groups compared to the NSS group (21.01%), potentially reflecting a shift toward other SCFA-producing taxa such as *Lactobacillus* and *Bacteroides*. In carcinogen-exposed rats, the (DEN + DMH) +ASBP-AHF group exhibited higher abundances of *Lactobacillus*, *Muribaculaceae*, *Christensenellaceae_R.7_group*, and *Lachnospiraceae_NK4A136_group*, all associated with gut barrier integrity and anti-inflammatory effects, indicating stronger microbiota restoration. Conversely, the (DEN + DMH) +ASBP-AHS group was enriched in *Bacteroides*, *Turicibacter*, *Romboutsia, Clostridia_UCG.014*, and *Marvinbryantia*, several of which are also known SCFA producers. Notably, *Bacteroides* remained dominant in ASBP-AHS-treated groups regardless of carcinogen exposure, which may contribute to the observed SCFA improvements. Meanwhile, *Enterorhabdus*, which was elevated in the DEN + DMH group, was reduced following ASBP-AHS and ASBP-AHF treatment, suggesting a partial reversal of carcinogen-induced dysbiosis.

Collectively, these findings suggest that both ASBP-AHS and ASBP-AHF attenuate carcinogen-induced gut dysbiosis by enhancing microbial diversity, restoring beneficial taxa, and shifting the microbial community structure toward a more balanced profile. While ASBP-AHF exhibited a slightly greater impact on overall microbial restoration, ASBP-AHS preferentially enriched SCFA-producing genera, highlighting its potential in promoting functional recovery of the gut microbiota.

### 2.6. Effect of ASBP-AHS and ASBP-AHF on SCFA Levels in DEN + DMH-Treated Rats

Gut dysbiosis and SCFA production are closely associated with CRC progression. To investigate whether ASBP-AHS and ASBP-AHF influence microbial metabolites, colonic SCFA concentrations were measured in DEN + DMH-treated rats. As shown in Table 4, DEN and DMH administration markedly decreased the levels of acetate, propionate, butyrate, valerate, and isovalerate compared to the NSS group. However, treatment with ASBP-AHS at 500 mg/kg body weight significantly increased acetate and propionate concentrations relative to the DEN + DMH group, restoring levels comparable to those in the NSS control. ASBP-AHF also promoted partial recovery of these SCFAs, although the changes were not statistically significant. Similarly, both ASBP-AHS and ASBP-AHF increased isobutyrate levels relative to the DEN + DMH group, but the differences were not significant. In contrast, butyrate levels remained relatively unchanged across all groups. These findings suggest that ASBP-AHS exerts a stronger modulatory effect on SCFA production than ASBP-AHF, particularly through the enhancement of acetate and propionate levels, which may support improved microbial activity and colonic health.

### 2.7. Correlation Between Gut Microbial Genera and SCFA Production Following ASBP-AHS and ASBP-AHF Intervention

**Figure 5:**
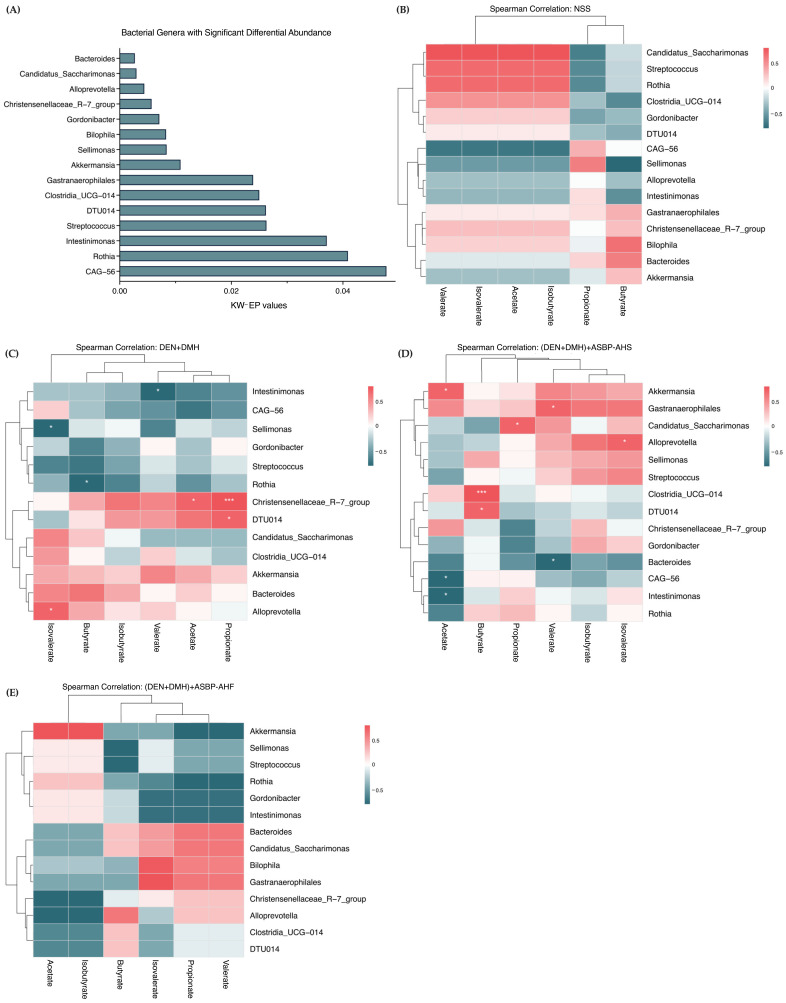
Correlation heatmaps and relative abundance of key gut microbiota across treatment groups. (**A**) Bacterial genera with significantly different abundances among groups, identified using the Kruskal–Wallis test with effect size estimation (KW-EP; *p* < 0.05). (**B**–**E**) Spearman correlation heatmaps showing associations between selected microbial genera and SCFA levels in each group. Red indicates positive correlations; dark green indicates negative correlations. Each group included *n* = 5 rats, except for the (DEN + DMH) + ASBP-AHF group (*n* = 4). The statistical differences were assessed using the Kruskal–Wallis test, followed by Wilcoxon rank-sum tests for pairwise comparisons. Significant correlations are indicated as * *p* < 0.05, *** *p* < 0.001. Abbreviations: normal saline solution (NSS); Diethylnitrosamine (DEN); 1,2-dimethylhydrazine (DMH); alkali-soluble BSFL protein (ASBP); alkali-soluble BSFL protein-Alcalase hydrolysate (spray-dried, ASBP-AHS; freeze-dried, ASBP-AHF).

To further investigate the functional relevance of microbial alterations, bacterial genera with significantly different abundances among treatment groups were identified using the Kruskal–Wallis test with effect size estimation (KW-EP; *p* < 0.05) (Data S1). A total of 15 genera were found to be significantly altered (Figure 5A). These genera were then selected for correlation analysis with SCFA concentrations to explore potential associations between gut microbiota composition and metabolite production. Spearman correlation heatmaps were used to assess associations between microbial genera and SCFA levels in the NSS and carcinogen-treated groups. In the NSS group, acetate, propionate, and isobutyrate levels were high (Table 4), indicating a healthy metabolic profile. *Candidatus_Saccharimonas*, *Streptococcus*, *Rothia*, and *Clostridia_UCG-014* showed strong positive correlations with acetate and isobutyrate, suggesting roles in their production. These genera were weakly or negatively correlated with propionate, while *Sellimonas* was positively associated with propionate, indicating a specific role in its synthesis (Figure 5B).

Following carcinogen exposure (Figure 5C), *Candidatus_Saccharimonas*, *Streptococcus*, *Rothia*, and *Clostridia_UCG-014* exhibited neutral or negative correlations with acetate and isobutyrate, reversing the patterns observed in the NSS group. In contrast, significant positive correlations were observed between *Christensenellaceae_R-7_group* and both propionate (*p* < 0.001) and acetate (*p* < 0.05), and between *DTU014* and propionate (*p* < 0.05). *Alloprevotella* was positively associated with isovalerate (*p* < 0.05). Negative correlations were found between *Sellimonas* and isovalerate, *Rothia* and butyrate, and *Intestinimonas* and valerate (*p* < 0.05), suggesting that DEN + DMH exposure disrupted normal microbiota–SCFA interactions, contributing to SCFA depletion.

ASBP-AHS treatment significantly increased acetate and propionate levels compared to the DEN + DMH group (Table 4). Correlation analysis showed positive associations between acetate and *Akkermansia* (*p* < 0.05), *Gastranaerophilales*, and *Christensenellaceae_R-7_group*. Similarly, *Candidatus_Saccharimonas* (*p* < 0.05), *Gastranaerophilales*, *Intestinimonas*, *Rothia*, and *Akkermansia* were positively correlated with propionate (Figure 5D). In contrast, *Candidatus_Saccharimonas*, *Intestinimonas*, and *Rothia* had shown negative correlations with propionate in the DEN + DMH group, indicating a reversal of dysregulated SCFA associations. These findings align with the increased relative abundances of *Candidatus_Saccharimonas*, *Gastranaerophilales*, and *Akkermansia* in the (DEN + DMH) + ASBP-AHS group. Additionally, *Candidatus_Saccharimonas*, *Gastranaerophilales*, *Alloprevotella*, and *Clostridia_UCG-014* were significantly enriched in the ASBP-AHS group, each exhibiting strong positive correlations with specific SCFAs: *Candidatus_Saccharimonas* with propionate (*p* < 0.05), *Gastranaerophilales* with valerate (*p* < 0.05), *Alloprevotella* with isovalerate (*p* < 0.05), and *Clostridia_UCG-014* with butyrate (*p* < 0.001). These results indicate that ASBP-AHS may enhance microbial functionality associated with SCFA production.

Compared to ASBP-AHS, the ASBP-AHF group showed a similar trend in microbial abundance and SCFA correlations (Figure 5E). *Akkermansia* and *Rothia* were positively correlated with acetate, while *Candidatus_Saccharimonas*, *Gastranaerophilales*, *Bacteroides*, and *Biophila* showed positive correlations with isobutyrate, propionate, and valerate. Although these associations suggest enhanced SCFA-related microbial activity, SCFA levels in the ASBP-AHF group were elevated relative to the DEN + DMH group but did not reach statistical significance.

Overall, these findings indicate that DEN + DMH exposure disrupted normal microbiota–SCFA interactions, contributing to gut dysbiosis. ASBP-AHS supplementation effectively restored SCFA levels and promoted beneficial microbial shifts, particularly by enhancing SCFA-associated taxa. While ASBP-AHF exhibited similar trends, its effects were less pronounced. These results suggest that ASBP-AHS has greater potential to counteract carcinogen-induced dysbiosis by supporting SCFA-producing bacteria and restoring microbial functionality.

### 2.8. Pyroglutamyl Peptides Identified in ASBP-AHS and ASBP-AHF

**Figure 6:**
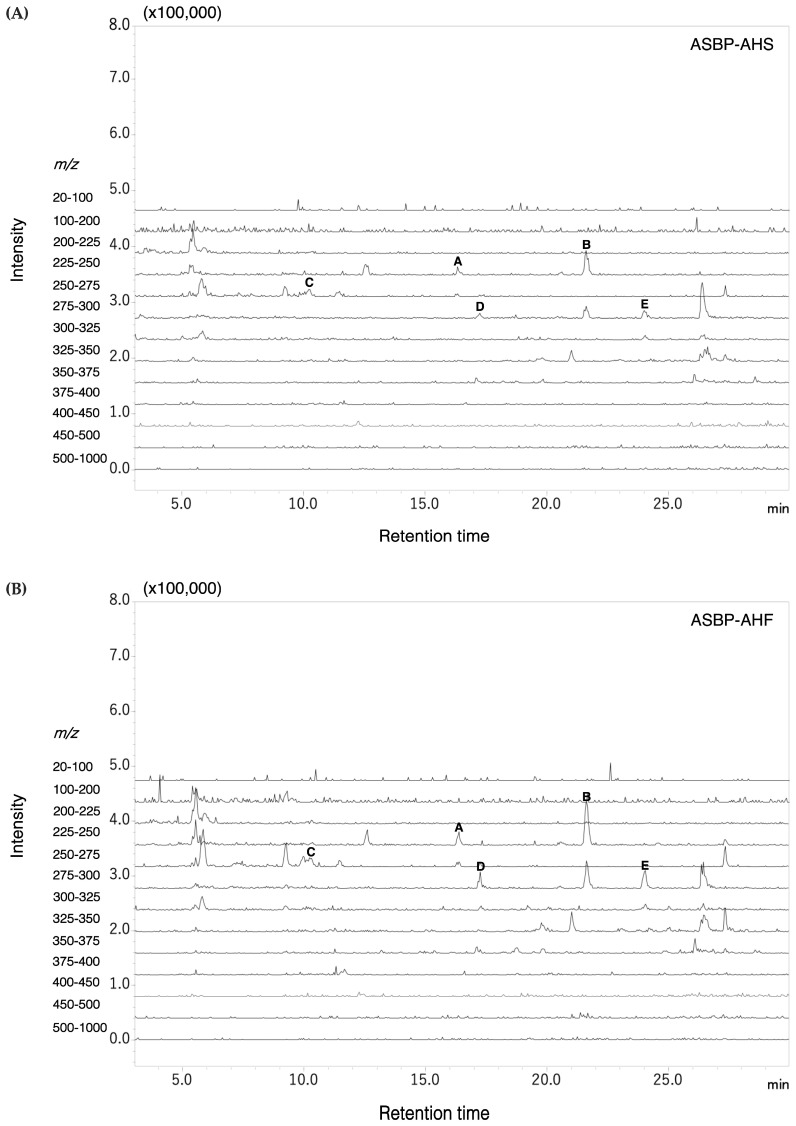
Representative LC-MS/MS chromatograms of pyroglutamyl peptides in (**A**) ASBP-AHS and (**B**) ASBP-AHF, acquired in total ion scan mode using positive electrospray ionization. The scan range was set to a mass-to-charge ratio (*m*/*z*) of 225–300. Peaks were identified as follows: (A) Pyroglutamyl-valine (pGlu-Val); (B) Pyroglutamyl-leucine (pGlu-Leu); (C) Pyroglutamyl-glutamic acid (pGlu-Glu); (D) Pyroglutamyl-tyrosine (pGlu-Tyr); and (E) Pyroglutamyl-phenylalanine (pGlu-Phe). Abbreviations: alkali-soluble BSFL protein-Alcalase hydrolysate (spray-dried, ASBP-AHS; freeze-dried, ASBP-AHF).

pGlu peptides, formed by cyclization of glutamine or glutamic acid during enzymatic hydrolysis [19], were identified in both ASBP-AHS and ASBP-AHF. Precursor ion scanning in the *m*/*z* range of 225–300 revealed identical peak patterns and *m*/*z* values in both hydrolysates. Representative ion chromatograms are shown in Figure 6. Liquid chromatography–tandem mass spectrometry (LC-MS/MS) analysis confirmed the presence of five di-pyroglutamyl peptides, with structures predicted based on precursor and fragment ion *m*/*z* values (Table 5). Several of these, including pGlu-Leu (pyroglutamyl-leucine), pGlu-Val (pyroglutamyl-valine), pGlu-Phe (pyroglutamyl-phenylalanine), and pGlu-Tyr (pyroglutamyl-tyrosine), have been associated with anti-inflammatory or gut-supportive effects. Notably, pGlu-Leu has been reported to alleviate dysbiosis by inducing host-derived antimicrobial peptide production. Their consistent detection and shared profiles suggest strong structural stability and potential functional relevance.

## 3. Discussion

CRC remains a leading cause of cancer-related mortality worldwide [20]. Recent studies have reported that bioactive peptides produced through enzymatic hydrolysis may help prevent colorectal carcinogenesis by modulating the gut microbiota and increasing SCFA production, thereby enhancing intestinal barrier integrity [11,21]. These peptides, commonly derived from food sources including insects, exhibit various biological activities such as antioxidant, anti-inflammatory, and anticancer effects [22]. Our previous study demonstrated that ASBP-AH, particularly its lowest molecular weight fraction, exhibited anticancer activity in COLO205 cells, likely due to its hydrophobic and charged amino acid composition [14]. However, its chemopreventive potential in vivo against colon cancer has not been investigated. In this study, ASBP-AH was further processed using spray-drying (ASBP-AHS) and freeze-drying (ASBP-AHF), two common techniques for preparing peptide-based ingredients [23]. Their efficacy was evaluated in a DEN + DMH-induced rat model of early-stage colorectal carcinogenesis, focusing on lesion development, inflammation, SCFA production, and gut microbiota modulation. Evaluation of physiological parameters, including food and water intake, body weight, relative organ weights, and serum transaminase levels (AST and ALT), demonstrated that ASBP and its hydrolysates, produced by Alcalase hydrolysis and either spray- or freeze-drying, did not elicit hepatotoxic effects. Nevertheless, co-administration with DEN + DMH did not mitigate the hepatic enzyme elevations, indicating limited hepatoprotective efficacy under the present experimental conditions.

DEN and DMH represent a well-established multiple-organ carcinogenicity model for evaluating chemopreventive agents in both liver and colorectal cancer. This model enables the assessment of test compounds in two target organs within a single animal over a relatively short experimental period [24,25]. However, the present study specifically focused on the chemopreventive effects of ASBP-AH in early-stage colorectal carcinogenesis; therefore, the analyses and discussion are limited to colon-associated outcomes. DMH is a potent colon-specific carcinogen widely used in rodent models. Following administration, DMH is metabolized in the liver into MAM and AOM, which are transported to the colon via the bloodstream or bile. In the colon, MAM is further converted into methyldiazonium ion, a reactive metabolite that induces DNA methylation, oxidative stress, and lipid peroxidation—ultimately promoting genomic instability, inflammation, and tumor initiation. DMH-induced colorectal tumorigenesis typically begins at the colonic crypts with the formation of ACF, which are widely recognized as early preneoplastic lesions [17]. In this study, high-dose ASBP-AHS and ASBP-AHF treatments significantly reduced ACF numbers, while ACF size remained unchanged, indicating a limited impact on lesion progression. The comparable extent of ACF reduction between the spray-dried and freeze-dried forms suggests that the drying method did not significantly influence chemopreventive efficacy. These results imply that both ASBP-AHS and ASBP-AHF primarily exert their effects by targeting mechanisms involved in the initiation and early stages of carcinogenesis. This may involve inhibition of DNA adduct formation, reduction in xenobiotic metabolizing enzyme activity, or modulation of inflammation [26]. Additionally, these compounds may influence cell proliferation or apoptosis, thereby preventing the formation of new ACF while having limited impact on existing lesions. Similar findings were reported with *Ficus dubia* latex extract, which reduced DMH-induced ACF numbers without affecting their size by modulating xenobiotic metabolism, inflammation, and cell turnover [27]. Likewise, Thai fermented soybean (Thua-Nao) has been shown to prevent early-stage colorectal carcinogenesis induced by DEN and DMH through the modulation of cell proliferation and gut microbiota in rats [24]. In our previous work, ASBP-AH also demonstrated anti-mutagenic and antioxidant properties, supporting its potential role in early chemoprevention [14].

Maintaining the balance between cell proliferation and apoptosis is essential for intestinal mucosal integrity. Disruption of this balance contributes to hyperplasia and carcinogenesis [28]. Therefore, inhibiting proliferation and promoting apoptosis are key strategies in cancer prevention. PCNA, a non-histone nuclear protein expressed during the cell cycle, serves as a well-established marker of cell proliferation and is typically upregulated during early tumorigenesis [29]. In this study, PCNA expression was significantly elevated in the DEN + DMH group, indicating enhanced colonic epithelial proliferation associated with increased ACF formation. Treatment with ASBP-AHS and ASBP-AHF significantly reduced PCNA levels. These effects are consistent with our previous in vitro findings, where the lowest molecular weight fraction of ASBP-AH inhibited proliferation in COLO205 cells via modulation of the SKP2/p21/cyclin D1 axis [14]. In contrast, cleaved caspase-3, a marker of apoptosis [30], showed no significant changes across groups, suggesting that the chemopreventive effects were not mediated through apoptosis induction. Inflammation also plays a critical role in tumor initiation and promotion [31]. COX-2, a key inflammatory marker, is upregulated during inflammation and has been linked to colon injury and polyp formation [32], making it a potential target for chemoprevention. Additionally, DMH has been shown to activate NF-κB, a master transcription factor that regulates inflammatory responses and promotes tumorigenesis, thereby contributing to inflammation-driven carcinogenesis [33]. In the present study, colonic tissues from DEN + DMH-treated rats exhibited marked upregulation of COX-2 and NF-κB expression, confirming inflammation-associated tumor promotion. Notably, both ASBP-AHS and ASBP-AHF significantly attenuated COX-2 and NF-κB levels, restoring them to near-control values. These findings suggest that ASBP-AHS and ASBP-AHF may exert chemopreventive effects, at least in part, through anti-proliferative and anti-inflammatory mechanisms.

Accumulating evidence indicates that gut microbiota plays a crucial role in maintaining intestinal homeostasis, while dysbiosis contributes to colorectal carcinogenesis [1]. In this study, DEN and DMH treatment significantly reduced microbial species richness and altered overall diversity, reflecting carcinogen-induced disruption of gut microbial balance. This finding is supported by numerous studies showing that DMH induces gut microbiota dysbiosis in colorectal cancer models [24,34]. In contrast, treatment with ASBP-AHS and ASBP-AHF restored species richness and shifted microbial diversity profiles closer to those of the healthy control group. These changes suggest that ASBP-derived peptides may counteract carcinogen-induced dysbiosis and help restore gut microbial homeostasis. In parallel, carcinogen exposure induced notable compositional shifts across taxonomic levels, further supporting the presence of gut dysbiosis. At the phylum level, the DEN + DMH group exhibited an elevated *Firmicutes*-to-*Bacteroidota* ratio and increased *Verrucomicrobiota* abundance—profiles associated with inflammation and microbial imbalance [35]. At the family level, both hydrolysate peptide treatments enriched beneficial taxa such as *Lactobacillaceae* and *Christensenellaceae*, which are known to support SCFA production, immune modulation, and gut barrier integrity [36,37]. At the genus level, ASBP-AHS promoted the abundance of *Bacteroides*, *Turicibacter*, *Romboutsia*, *Clostridia_UCG-014*, and *Marvinbryantia*, enhancing microbial functions related to SCFA production [38,39,40,41,42]. In contrast, ASBP-AHF favored the enrichment of genera linked to anti-inflammatory activity and mucosal protection, including *Lactobacillus*, *Muribaculaceae*, *Christensenellaceae_R-7_group*, and *Lachnospiraceae_NK4A136_group* [43,44,45,46]. Additionally, both treatments reduced harmful or pro-inflammatory genera such as *Enterorhabdus* [47], indicating a partial reversal of carcinogen-induced microbial disturbances. Collectively, these findings suggest that ASBP-AHS and ASBP-AHF contribute to the restoration of gut microbial balance by promoting beneficial taxa and suppressing dysbiotic ones, with ASBP-AHS more strongly associated with SCFA-producing bacteria, and ASBP-AHF favoring anti-inflammatory microbial signatures.

Recent studies have shown that alterations in the gut microbiota can reduce SCFA production in the colon, thereby impacting intestinal barrier function and immune regulation—factors closely linked to CRC development and progression [10]. SCFAs such as acetate, propionate, and butyrate are produced through microbial fermentation of dietary fibers and non-digestible carbohydrates. These metabolites not only support gut health but also selectively inhibit the proliferation of colorectal cancer cells without harming normal cells [48]. To evaluate the functional relevance of microbial shifts, 15 differentially abundant genera were analyzed for their correlations with SCFA levels. In the DEN + DMH group, *Candidatus_Saccharimonas*, *Streptococcus*, *Rothia*, and *Clostridia_UCG-014* showed negative correlations with acetate and isobutyrate, suggesting a disruption of SCFA-associated microbial functionality. Notably, *Streptococcus* and *Clostridia* are known for their roles in carbohydrate fermentation and SCFA production [49]. ASBP-AHS treatment notably restored the relative abundance of *Candidatus_Saccharimonas*, *Gastranaerophilales*, and *Akkermansia*—taxa that exhibited significant positive correlations with acetate and propionate levels, both of which were significantly elevated following intervention. These results indicate that the re-establishment of these microbial populations likely contributed to the recovery of acetate and propionate production. In contrast, *Clostridia_UCG-014* also demonstrated a significant positive correlation with butyrate; however, its relative abundance was comparatively lower than that of the acetate- and propionate-associated taxa. This may partially explain the lack of a significant increase in butyrate content, suggesting that the abundance of butyrate-producing microbes remained insufficient to enhance butyrate levels. The observed positive correlations between *Akkermansia* and SCFA levels are likely linked to its role in mucin degradation, which enhances acetate production and supports intestinal barrier integrity [50]. Similarly, *Gastranaerophilales* may contribute to microbial interactions that influence SCFA availability [51]. While *Candidatus_Saccharimonas* showed a positive correlation with propionate, its specific metabolic role in SCFA production remains unclear. In contrast, ASBP-AHF treatment showed positive correlations between *Akkermansia* and *Rothia* with acetate, and between *Candidatus_Saccharimonas*, *Gastranaerophilales*, *Bacteroides*, and *Biophila* with isobutyrate, propionate, and valerate. However, SCFA levels in the ASBP-AHF group were not significantly different from those in the carcinogen-treated group. These findings suggest that ASBP-AHS more effectively restores SCFA-producing microbial activity, while ASBP-AHF may contribute to microbial–metabolite interactions with comparatively lower impact on SCFA levels.

pGlu peptides, characterized by a cyclic pyroglutamate residue at the N-terminus, are formed either naturally or through enzymatic hydrolysis of glutamine- or glutamic acid-containing peptides [19]. These modifications enhance peptide stability by protecting against exopeptidase degradation, potentially prolonging their activity in the gastrointestinal tract [52]. Increasing evidence has highlighted pGlu-containing peptides for their diverse biological properties in both in vitro and in vivo studies [19]. Peptidomic analysis identified five pGlu peptides in both hydrolysates following simulated gastrointestinal digestion, including pGlu-Val, pGlu-Phe, and pGlu-Tyr—several of which have been associated with anti-inflammatory and gut-supportive functions [53,54]. Recent studies further suggest that pGlu peptides can modulate host–microbiota interactions by influencing microbial composition and host immune responses. Notably, pGlu-Leu has been shown to stimulate the expression of host-derived antimicrobial peptides, thereby contributing to the rebalancing of dysbiotic microbiota [11,55]. The consistent detection of these pGlu peptides in ASBP-AHS and ASBP-AHF following gastrointestinal digestion suggests that they remain stable during sample preparation and digestive processes, likely owing to the high structural stability conferred by the pyroglutamic acid modification at the N-terminus [19]. Although high inlet temperatures can increase the risk of thermal degradation, conditions ranging from 130 to 190 °C with outlet temperatures below 100 °C are generally considered suitable for preserving bioactive compounds during spray drying [56]. In this study, the use of a 190 °C inlet temperature and an 80 °C outlet temperature, together with the short residence time and the protective effect of maltodextrin, likely helped maintain peptide integrity and bioactivity in the ASBP-AHS group. Since dietary peptides are generally not absorbed systemically in significant amounts, their functional effects are more likely to occur through local interactions within the gut, including modulation of microbiota composition and SCFA production [57]. Spray-drying may enhance peptide functionality by improving dispersion and accessibility in the gut, thereby supporting greater microbial interaction and SCFA production. Both the spray-dried and freeze-dried forms improved gut dysbiosis by enhancing microbial diversity and restoring beneficial taxa, likely because they share a similar composition of pGlu peptides. However, ASBP-AHS more effectively enriched SCFA-producing genera and increased total SCFA levels, which may be partly attributed to the presence of maltodextrin introduced during the spray-drying process. As maltodextrin is fermentable by gut microbes, its inclusion can promote the growth of beneficial bacteria and further enhance SCFA production [58]. These findings highlight the importance of processing methods in modulating peptide–microbiota interactions and gut bioactivity.

## 4. Materials and Methods

### 4.1. Chemicals

Alcalase (3.018 U/mL) was obtained from Merck (Darmstadt, Germany). DEN and 3,3′-diaminobenzidine (DAB) were purchased from Sigma-Aldrich (St. Louis, MO, USA). DMH was sourced from TCI (Tokyo, Japan). The avidin–biotin horseradish peroxidase complex (ABC) kit and the Vectastain^®^ Elite ABC Kit (Universal) were obtained from Vector Laboratories Inc. (Burlingame, CA, USA). All other chemicals used were of analytical grade.

### 4.2. Preparation of Samples

ASBP-AH was prepared according to the method described by Praseatsook et al. [14]. The enzymatic reaction was terminated by heating at 90 °C for 10 min, followed by centrifugation at 6000 rpm for 15 min. The supernatant was collected and subjected to two drying methods. For freeze-drying, the sample was stored at −20 °C prior to lyophilization, yielding ASBP-AHF. For spray-drying, maltodextrin (10% *w*/*v*) was added to the supernatant, and the mixture was processed using a spray dryer (inlet temperature: 190 °C; outlet temperature: 80 °C; feed rate: 1 mL/min) to obtain ASBP-AHS.

### 4.3. Animals and Experimental Protocol

Three-week-old male Wistar rats (90–100 g) were obtained from Nomura Siam International Co., Ltd. (Bangkok, Thailand) and housed at the Animal House, Faculty of Medicine, Chiang Mai University (Chiang Mai, Thailand). The animals were maintained under standard environmental conditions (25 °C, 12 h light/dark cycle) and provided ad libitum access to a commercial basal diet (C.P. Mice Feed 082G, Samut Prakan, Thailand), with nutritional composition as described previously [24], and tap water. The experimental protocol was approved by the Animal Ethics Committee of the Faculty of Medicine, Chiang Mai University (Protocol No. 20/2565, approved on 8 September 2022). All procedures adhered to institutional guidelines and the ARRIVE reporting standards.

**Figure 7:**
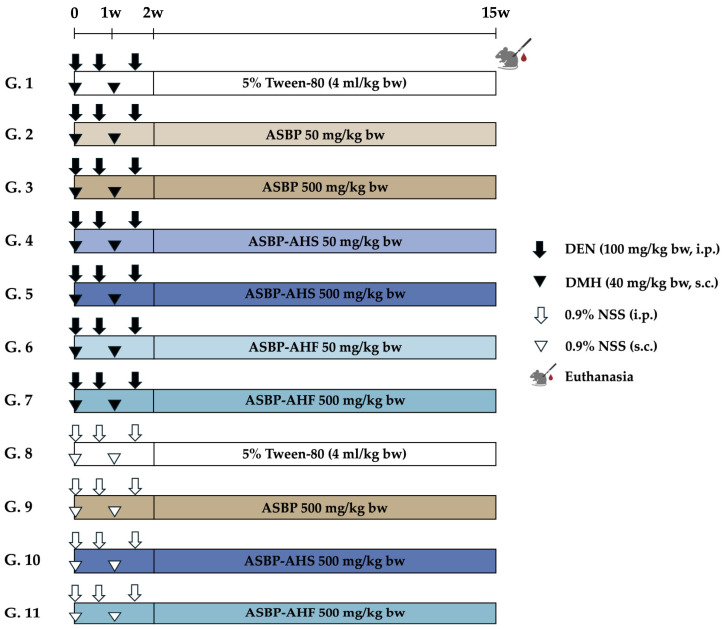
Experimental protocol for evaluating the chemopreventive potential of ASBP, ASBP-AHS, and ASBP-AHF—during the early stage of colon carcinogenesis in DEN- and DMH-induced rats. Abbreviations: normal saline solution (NSS); Diethylnitrosamine (DEN); 1,2-dimethylhydrazine (DMH); alkali-soluble BSFL protein (ASBP); alkali-soluble BSFL protein-Alcalase hydrolysate (spray-dried, ASBP-AHS; freeze-dried, ASBP-AHF); intraperitoneal (i.p.); subcutaneous (s.c.).

The experiment was conducted over a 15-week period (Figure 7). A total of 11 groups (*n* = 8 per group) were randomly assigned to different treatment regimens. Groups 1–7 received carcinogenic doses of DEN (100 mg/kg body weight [bw], i.p.) on days 0, 4, and 11, and DMH (40 mg/kg bw, s.c.) on days 0 and 7. Following carcinogen administration, rats were treated via oral gavage five days per week for 13 weeks. All test samples were dissolved in 5% Tween 80. Group 1 received only 5% Tween 80 and served as the vehicle control. Groups 2 and 3 were treated with ASBP at 50 and 500 mg/kg body weight (bw), respectively. Groups 4 and 5 received ASBP-AHS at the same doses, while Groups 6 and 7 received ASBP-AHF. Groups 8–11 were injected with 0.9% NSS instead of carcinogens and served as additional control and treatment groups. Group 8 received 5% Tween 80 and was designated as the negative control group, while Groups 9–11 received ASBP, ASBP-AHS, or ASBP-AHF at 500 mg/kg bw, respectively. Throughout the experiment, body weight, food intake, and water intake were recorded weekly to monitor general health and treatment effects. At the end of the study, rats were euthanized by isoflurane overdose. Blood samples were collected for aspartate AST and ALT analysis at the Small Animal Hospital, Faculty of Veterinary Medicine, Chiang Mai University. The liver, spleen, and kidneys were excised and weighed. The colon was perfused with 10% neutral buffered formalin, chilled on ice, and divided into proximal, distal, and rectal segments for ACF evaluation. Colonic tissues were embedded in paraffin for immunohistochemical analysis. Fecal samples were collected directly from the anus and stored at −80 °C for microbiota composition and SCFA analysis.

### 4.4. Determination of Preneoplastic Lesions in Colon Tissues

ACF were analyzed using methylene blue staining. After fixation in formalin, the colons were opened longitudinally along the median axis and stained with 2% methylene blue. The total number of crypts and the number of crypts per focus were assessed under a light microscope for each rat [24].

### 4.5. Effects of ASBP-AHS and ASBP-AHF on Cell Proliferation, Apoptosis, and Pro-Inflammatory

#### Cytokine Expression in Colon Tissues

Deparaffinized colon sections were subjected to antigen retrieval in sodium citrate buffer (pH 6.0) at elevated temperature, followed by incubation with 3% hydrogen peroxide to block endogenous peroxidase activity. The sections were then incubated overnight at 4 °C with primary antibodies at the following dilutions: PCNA (1:500; mouse monoclonal antibody, DAKO, M0879, Glostrup, Denmark), cleaved caspase-3 (1:100; rabbit monoclonal antibody, Cell Signaling Technology, #9664, Danvers, MA, USA), NF-κB (1:200; mouse monoclonal antibody, Cell Signaling Technology, #6956, Danvers, MA, USA), and COX-2 (1:500; rabbit monoclonal antibody, Cell Signaling Technology, #12282, Danvers, MA, USA). Immunoreactivity was detected using biotin-labeled goat anti-rabbit immunoglobulin G, followed by the application of the ABC kit and DAB substrate. Staining was visualized under a light microscope. The immunohistochemistry procedure was adapted from a previously published protocol [24].

### 4.6. Immunohistochemistry Image Analysis and Histological Score Quantification

The images of NF-κB and COX-2 immunostaining were captured from five randomly selected high-power fields (400×) per section for histological score (H-score) analysis. Quantification of staining intensity was performed using ImageJ software (v1.53t, NIH, Bethesda, MD, USA). The images were converted to 8-bit grayscale and processed using the built-in “H DAB” color deconvolution function to isolate the DAB signal. The Colour_2 (DAB) channel was used for analysis. Thresholds were applied to classify staining intensity as strong (0–130), moderate (131–180), or weak (181–255) based on pixel values. The percentage of stained area in each category was measured using the “Analyze → Measure” function, with “Limit to threshold” and “Area fraction” options enabled [59,60,61]. H-scores were calculated using the following formula:

H-score = (% Weak × 1) + (% Moderate × 2) + (% Strong × 3)

Yielding a total possible score ranging from 0 to 300 [62]. The quantification was performed separately for NF-κB and COX-2 expression.

### 4.7. Measurement of SCFAs in Rat Feces

SCFAs were extracted from fecal samples using a modified method described by Phannasorn et al. [63]. Fecal samples were collected from all experimental animals; however, only four samples were available in the (DEN + DMH) + ASBP-AHF group, as it was not possible to obtain sufficient feces from one animal. Five samples were collected in all other groups. The statistical analyses confirmed that this difference in sample size did not compromise the validity of the findings. Briefly, 500 mg of frozen fecal material was homogenized in sterile water and treated with 25% metaphosphoric acid to precipitate proteins. After centrifugation, the supernatant was filtered through a 0.45 µm nylon membrane filter. SCFAs were analyzed using a SCION 436-GC system (BRUKER, Billerica, MA, USA) equipped with a flame ionization detector and a Restek™ RTx-1 capillary column (15 m × 0.53 mm ID, 5 µm film thickness). Acetate, propionate, butyrate, isobutyrate, valerate, and isovalerate were quantified using external calibration curves and expressed as µmol/g feces.

### 4.8. Analysis of Composition of Fecal Intestinal Microbiota in Rat

The composition of the intestinal microbiota was analyzed via 16S rRNA gene amplicon sequencing. Genomic DNA was extracted from fecal samples using the QIAamp DNA Microbiome Kit (Qiagen, Hilden, Germany), following the protocol described by Taya et al. [24]. The V3–V4 hypervariable regions of the bacterial 16S rRNA gene were amplified using the universal primer pair (Forward: 5′-TCGTCGGCAGCGTCAGATGTGTATAAGAGACAGCCTACGGGNGGCWGCAG-3′; Reverse: 5′-GTCTCGTGGGCTCGGAGATGTGTATAAGAGACAGGACTACHVGGGTATCTAATCC-3′) according to the Illumina 16S Metagenomics protocol. Amplicon libraries were sequenced with paired-end reads (2 × 300 bp) on the Illumina MiSeq platform (Illumina, San Diego, CA, USA). To validate taxonomic accuracy and methodological reliability, a mock microbial community standard was included in each sequencing batch. The sequence data were processed using QIIME2 (v2023.7.0) for quality filtering, denoising, read merging, and chimera removal. Taxonomic classification was performed with the SILVA 16S rRNA reference database (release 138), and further analyses were conducted in R (v4.3.2).

Genus-level relative abundance was calculated as the percentage of total reads per sample. Taxa with a mean relative abundance below 0.01% across all samples were excluded from further analysis. Alpha diversity was estimated using the Chao1 index, while beta diversity was assessed based on UniFrac phylogenetic distances. The statistical comparisons of genus-level abundance between groups were performed using the Kruskal–Wallis test, followed by pairwise Wilcoxon rank-sum tests, with significance set at *p* < 0.05.

Spearman’s rank correlation analysis was used to assess associations between bacterial genera and fecal SCFA concentrations. Only genera exhibiting statistically significant differences among groups were included in the correlation analysis. Taxa or metabolites with no variability or near-zero values were excluded to ensure statistical robustness. Correlation heatmaps were generated using the pheatmap package in R (version 4.3.2), with a custom diverging color palette applied to visualize correlation coefficients and corresponding significance levels.

### 4.9. Determination of the Bioactive Peptides Using LC-MS/MS

To mimic gastrointestinal digestion and break down large peptides, intestinal peptidases were extracted from rat small intestinal mucosal cells using the method described by Wijanarti et al. [64]. For digestion, 20 µL of enzyme extract was mixed with 80 µL of the sample and incubated at 37 °C for 4 h. The reaction was terminated by adding ethanol at a volume three times that of the reaction mixture, followed by centrifugation at 120 rpm and 4 °C for 15 min. The resulting supernatant was collected, stored at −20 °C, and used for pGlu peptide determination via LC-MS/MS, as described by Pharapirom et al. [65].

### 4.10. Statistical Analysis

All results are expressed as mean ± standard deviation (SD). Group comparisons were performed using one-way analysis of variance (ANOVA), followed by Tukey’s post hoc test, using GraphPad Prism version 10.3.1 (GraphPad Software, Boston, MA, USA). For SCFA content, Duncan’s multiple range test was applied to detect significant differences among groups. A *p*-value < 0.05 was considered statistically significant. For microbiota-related data, alpha diversity differences among groups were assessed using the Kruskal–Wallis test, with pairwise comparisons performed using the Wilcoxon rank-sum test. Beta diversity variation was evaluated using permutational multivariate analysis of variance (PERMANOVA), as described in Section 2.8. Correlations between bacterial genera and fecal SCFA concentrations were analyzed using Spearman’s rank correlation.

## 5. Conclusions

In summary, both ASBP-AHS and ASBP-AHF protected against early-stage colorectal carcinogenesis by reducing ACF formation, suppressing PCNA, NF-κB, and COX-2 expression, and restoring gut microbial diversity. Stable pGlu peptides identified in both hydrolysates may contribute to anti-inflammatory and microbiota-modulating effects. Both treatments also promoted SCFA-associated microbes and partially restored SCFA levels, with stronger effects in ASBP-AHS. These findings highlight the potential of ASBP-AH, in either drying form, as a functional food ingredient for gut health and colorectal cancer prevention. Further studies should focus on isolating key bioactive peptides, clarifying their mechanisms of action, and evaluating the long-term safety and efficacy of ASBP-AH for human consumption and clinical applications.

## Figures and Tables

**Table 1:** Effects of ASBP, ASBP-AHS, and ASBP-AHF on body weight, food intake, and water consumption in rats treated with DEN and DMH.

Groups	Treatments	Body Weight (g)		Consumption (g/Day)	
		Initial	Final	Food	Water
1	DEN + DMH	123.33 ± 12.11	482.50 ± 33.87	20.71 ± 2.43	25.63 ± 3.67
2	(DEN + DMH) + ASBP 50 mg/kg bw	120.63 ± 7.29	469.38 ± 24.99	19.13 ± 2.20	25.25 ± 3.56
3	(DEN + DMH) + ASBP 500 mg/kg bw	124.38 ± 5.63	458.75 ± 29.73	19.17 ± 2.17	26.08 ± 3.53
4	(DEN + DMH) + ASBP-AHS 50 mg/kg bw	121.25 ± 6.94	461.88 ± 37.60	19.38 ± 2.28	25.28 ± 2.74
5	(DEN + DMH) + ASBP-AHS 500 mg/kg bw	117.50 ± 8.86	466.88 ± 46.13	19.54 ± 2.16	25.27 ± 3.31
6	(DEN + DMH) + ASBP-AHF 50 mg/kg bw	118.00 ± 7.58	447.00 ± 40.87	19.56 ± 2.61	25.16 ± 3.28
7	(DEN + DMH) + ASBP-AHF 500 mg/kg bw	125.83 ± 7.36	475.83 ± 41.76	20.00 ± 2.75	25.25 ± 3.48
8	NSS	127.86 ± 8.59	500.00 ± 32.79	19.92 ± 2.03	26.42 ± 5.02
9	ASBP 500 mg/kg bw	127.00 ± 11.51	452.00 ± 44.0	18.86 ± 2.07	25.16 ± 2.28
10	ASBP-AHS 500 mg/kg bw	133.00 ± 8.37	483.00 ± 34.2	19.42 ± 2.39	25.76 ± 3.75
11	ASBP-AHF 500 mg/kg bw	134.00 ± 6.52	487.00 ± 30.1	19.56 ± 3.50	25.97 ± 6.19

The values are presented as the mean ± SD. The statistical differences among groups were analyzed using one-way analysis of variance (ANOVA), followed by Tukey’s post hoc test for multiple comparisons. No statistically significant differences were observed (*p* > 0.05). Abbreviations: normal saline solution (NSS); Diethynitrosamine (DEN); 1,2-dimethylhydrazine (DMH); alkali-soluble BSFL protein (ASBP); alkali-soluble BSFL protein-Alcalase hydrolysate (spray-dried, ASBP-AHS; freeze-dried, ASBP-AHF).

**Table 2:** Effects of ASBP, ASBP-AHS, and ASBP-AHF on organ weights in rats treated with DEN and DMH.

Groups	Treatments	Liver		Kidney		Spleen	
		Absolute (g)	Relative (%)	Absolute (g)	Relative (%)	Absolute (g)	Relative (%)
1	DEN + DMH	13.98 ± 1.42	2.89 ± 0.16	2.95 ± 0.29	0.61 ± 0.06	0.85 ± 0.07	0.18 ± 0.02
2	(DEN + DMH) + ASBP 50 mg/kg bw	13.11 ± 0.54	2.80 ± 0.20	2.71 ± 0.26	0.58 ± 0.05	0.83 ± 0.13	0.18 ± 0.03
3	(DEN + DMH) + ASBP 500 mg/kg bw	12.88 ± 0.56	2.82 ± 0.17	2.75 ± 0.19	0.60 ± 0.03	0.84 ± 0.07	0.18 ± 0.02
4	(DEN + DMH) + ASBP-AHS 50 mg/kg bw	13.18 ± 1.24	2.86 ± 0.22	2.79 ± 0.28	0.60 ± 0.06	0.85 ± 0.09	0.19 ± 0.02
5	(DEN + DMH) + ASBP-AHS 500 mg/kg bw	12.83 ± 1.48	2.75 ± 0.13	2.73 ± 0.30	0.59 ± 0.04	0.84 ± 0.17	0.18 ± 0.04
6	(DEN + DMH) + ASBP-AHF 50 mg/kg bw	12.88 ± 2.06	2.87 ± 0.28	2.67 ± 0.18	0.60 ± 0.05	0.74 ± 0.08	0.17 ± 0.02
7	(DEN + DMH) + ASBP-AHF 500 mg/kg bw	12.70 ± 1.05	2.67 ± 0.07	2.85 ± 0.41	0.60 ± 0.05	0.75 ± 0.10	0.16 ± 0.01
8	NSS	12.72 ± 1.06	2.55 ± 0.24	2.85 ± 0.48	0.57 ± 0.07	0.74 ± 0.09	0.15 ± 0.01
9	ASBP 500 mg/kg bw	12.88 ± 0.56	2.82 ± 0.17	2.75 ± 0.19	0.60 ± 0.03	0.84 ± 0.07	0.18 ± 0.02
10	ASBP-AHS 500 mg/kg bw	12.36 ± 0.98	2.56 ± 0.07	2.71 ± 0.18	0.56 ± 0.03	0.69 ± 0.05	0.14 ± 0.01
11	ASBP-AHF 500 mg/kg bw	12.41 ± 1.31	2.54 ± 0.13	2.54 ± 0.14	0.52 ± 0.02	0.71 ± 0.09	0.15 ± 0.03

The values are presented as the mean ± SD. The statistical differences among groups were analyzed using one-way analysis of variance (ANOVA), followed by Tukey’s post hoc test for multiple comparisons. No statistically significant differences were observed (*p* > 0.05). Abbreviations: normal saline solution (NSS); Diethynitrosamine (DEN); 1,2-dimethylhydrazine (DMH); alkali-soluble BSFL protein (ASBP); alkali-soluble BSFL protein-Alcalase hydrolysate (spray-dried, ASBP-AHS; freeze-dried, ASBP-AHF).

**Table 3:** Effects of ASBP, ASBP-AHS, and ASBP-AHF treatments on serum AST and ALT levels in rats.

Groups	Treatments	AST (U/L)	ALT (U/L)
1	DEN + DMH	151.17 ± 41.52	66.83 ± 18.91 **
2	(DEN + DMH) + ASBP 50 mg/kg bw	133.13 ± 36.73	44.75 ± 9.02
3	(DEN + DMH) + ASBP 500 mg/kg bw	134.75 ± 39.12	58.25 ± 13.36 *
4	(DEN + DMH) + ASBP-AHS 50 mg/kg bw	146.75 ± 35.75	68.50 ± 24.05 ***
5	(DEN + DMH) + ASBP-AHS 500 mg/kg bw	132.75 ± 29.95	59.75 ± 14.61 *
6	(DEN + DMH) + ASBP-AHF 50 mg/kg bw	158.40 ± 46.15 *	76.00 ± 27.16 ***
7	(DEN + DMH) + ASBP-AHF 500 mg/kg bw	114.67 ± 44.32	47.67 ± 18.96
8	NSS	89.57 ± 23.39	29.86 ± 3.48
9	ASBP 500 mg/kg bw	78.40 ± 6.11	32.40 ± 1.52
10	ASBP-AHS 500 mg/kg bw	79.20 ± 3.63	27.00 ± 2.12
11	ASBP-AHF 500 mg/kg bw	73.60 ± 4.56	31.60 ± 3.21

The values are presented as the mean ± SD. The statistical analysis was performed using one-way ANOVA followed by Tukey’s post hoc test for multiple comparisons. * *p* < 0.05, ** *p* < 0.01, *** *p* < 0.001 vs. NSS group. Abbreviations: normal saline solution (NSS); Diethynitrosamine (DEN); 1,2-dimethylhydrazine (DMH); alkali-soluble BSFL protein (ASBP); alkali-soluble BSFL protein-Alcalase hydrolysate (spray-dried, ASBP-AHS; freeze-dried, ASBP-AHF).

**Table 4:** SCFA levels in the feces of DEN- and DMH-induced rats after ASBP-AHS and ASBP-AHF administration.

SCFA (µmol/g Feces)	Treatments			
	NSS	DEN + DMH	(DEN + DMH) + ASBP-AHS 500 mg/kg bw	(DEN + DMH) + ASBP-AHF 500 mg/kg bw
Acetate	40.42 ± 15.98 ^a^	10.11 ± 11.32 ^b^	39.12 ± 3.34 ^a^	18.71 ± 7.34 ^b^
Propionate	63.38 ± 3.41 ^a^	8.04 ± 5.35 ^c^	39.31 ± 5.91 ^b^	6.15 ± 4.34 ^c^
Butyrate	16.19 ± 0.07 ^a^	4.00 ± 0.26 ^b^	5.70 ± 2.46 ^b^	5.44 ± 1.72 ^b^
Isobutyrate	37.04 ± 20.06 ^a^	6.15 ± 7.43 ^b^	13.67 ± 0.45 ^ab^	17.12 ± 7.90 ^ab^
Isovalerate	4.91 ± 3.53 ^a^	1.28 ± 0.11 ^ab^	2.70 ± 1.23 ^ab^	0.98 ± 0.43 ^b^
Valerate	17.53 ± 5.65 ^a^	2.61 ± 0.88 ^b^	10.77 ± 7.07 ^ab^	3.01 ± 2.83 ^b^

The results are presented as mean ± SD. The statistical significance was determined using one-way ANOVA followed by Duncan’s multiple range test. Different superscript letters indicate significant differences between groups (*p* < 0.05). *n* = 5 rats per group, except for the (DEN + DMH) + ASBP-AHF group (*n* = 4). SCFA, short-chain fatty acids. Abbreviations: normal saline solution (NSS); Diethynitrosamine (DEN); 1,2-dimethylhydrazine (DMH); alkali-soluble BSFL protein (ASBP); alkali-soluble BSFL protein-Alcalase hydrolysate (spray-dried, ASBP-AHS; freeze-dried, ASBP-AHF).

**Table 5:** Identification of pyroglutamyl peptides in ASBP-AHS and ASBP-AHF by LC-MS/MS analysis.

Position	Pyroglutamyl Peptides	Precursor Ion (*m*/*z*)	Retention Time (min)	Precursor Scan (*m*/*z*)
A	Pyroglutamyl-valine (pGlu-Val)	229	16.3	72 (* Val), 84 (* pGlu), 118 (y1), 183 (a2), 229
B	Pyroglutamyl-leucine (pGlu-Leu)	243	22	84 (* pGlu), 86 (* Leu), 132 (y1), 197 (a2), 243
C	Pyroglutamyl-glutamic acid (pGlu-Glu)	259	10	84 (* pGlu), 102 (* Glu), 148 (y1), 241 (b1), 259
D	Pyroglutamyl-tyrosine (pGlu-Tyr)	293	17	84 (* pGlu), 136 (* Tyr), 182 (y1), 292 (y2 + ACN)
E	Pyroglutamyl-phenylalanine (pGlu-Phe)	277	24	84 (* pGlu), 120 (* Phe), 166 (y1), 231 (a2), 276 (y2)

* Represents immonium ion. ACN = acetonitrile.

## Data Availability

The original contributions presented in this study are included in the article and Supplementary Materials. Further inquiries can be directed to the corresponding author.

## Supplementary Materials

The following supporting information can be downloaded at https://www.mdpi.com/article/10.3390/ijms26135955/s1.

## Abbreviations

The following abbreviations are used in this manuscript:

ACF	Aberrant crypt foci
ACN	Acetonitrile
ASBP	Alkali-soluble Black Soldier Fly larvae protein
ASBP-AH	ASBP–Alcalase hydrolysate
ASBP-AHF	ASBP-AH prepared by freeze-drying
ASBP-AHS	ASBP-AH prepared by spray-drying
COX-2	Cyclooxygenase-2
DEN	Diethylnitrosamine
DMH	1,2-Dimethylhydrazine
IHC	Immunohistochemistry
LC-MS/MS	Liquid chromatography-tandem mass spectrometry
NF-κB	Nuclear factor kappa B
PCNA	Proliferating cell nuclear antigen
pGlu	Pyroglutamyl
pGlu-Glu	Pyroglutamyl-glutamic acid
pGlu-Leu	Pyroglutamyl-leucine
pGlu-Phe	Pyroglutamyl-phenylalanine
pGlu-Tyr	Pyroglutamyl-tyrosine
pGlu-Val	Pyroglutamyl-valine
SCFA	Short-chain fatty acid
