# Supplementary material for: Chemopreventive Effects of Bioactive Peptides Derived from Black Soldier Fly Larvae Protein Hydrolysates in a Rat Model of Early-Stage Colorectal Carcinogenesis
Source: Int J Mol Sci. 2025 Jun 20;26(13):5955. doi: 10.3390/ijms26135955 (PMC12249673; doi:10.3390/ijms26135955)

Supplementary Figure S1. No detectable cleaved caspase-3–positive cells in colon tissue sections across all experimental groups.

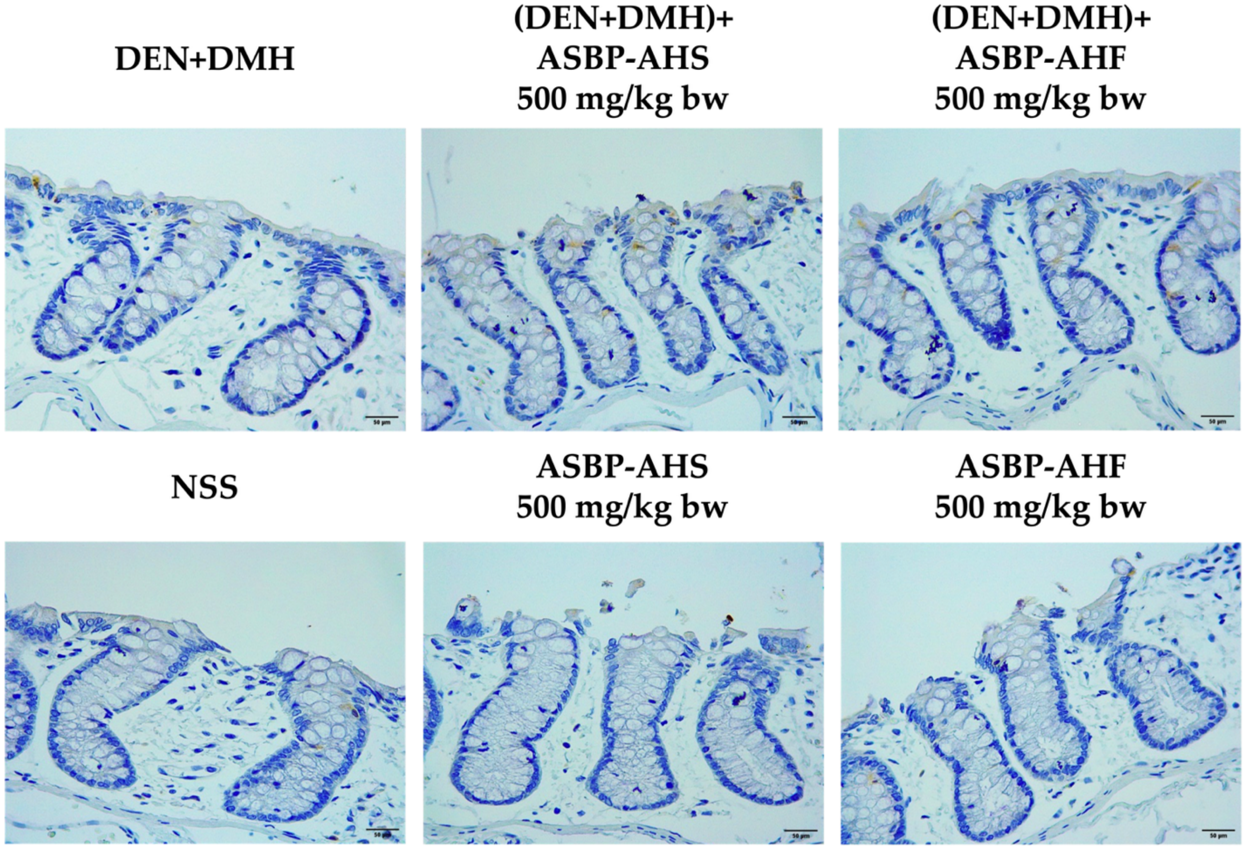

Supplement: Supplementary file 1 [file ijms-26-05955-s001.zip › Figure S1. IHC staining of cleaved caspase-3 in colonic tissues.pdf]
